# Neutrophil Cells Are Essential for The Efficacy of a Therapeutic Vaccine against Paracoccidioidomycosis

**DOI:** 10.3390/jof7060416

**Published:** 2021-05-26

**Authors:** Lucas dos Santos Dias, Leandro B. R. Silva, Joshua D. Nosanchuk, Carlos Pelleschi Taborda

**Affiliations:** 1 Department of Microbiology, Institute of Biomedical Sciences, University of São Paulo, São Paulo 05508-000, Brazil; dossantosdia@wisc.edu (L.d.S.D.); leandrobr87@usp.br (L.B.R.S.); 2 Departments of Medicine (Division of Infectious Diseases), Microbiology and Immunology, Albert Einstein College of Medicine, New York, NY 10461, USA; josh.nosanchuk@einsteinmed.org; 3 Laboratory of Medical Mycology, Tropical Medicine Institute USP-LIM53, University of São Paulo, São Paulo 05403-000, Brazil

**Keywords:** neutrophils 1, P10 2, vaccine 3, DODAB 4, *paracoccidioides brasiliensis* 5, paracoccidioidomycosis 6, neutrophil depletion 7

## Abstract

Paracoccidioidomycosis (PCM), caused by the *Paracoccidioides* species, is a systemic disease endemic in several Latin American countries, mainly in Brazil, Colombia, Argentina, and Venezuela. Current treatment approaches are challenging as they require prolonged durations of antifungal drugs that have potential toxicities, and despite antifungals, relapses are common. Hence, new therapeutic approaches, such as vaccines, are being investigated. The therapeutic vaccine consisting of peptide P10 associated with lipid cationic DODAB (P10+DODAB) is effective in murine models of PCM. However, the specific immune mechanisms required for the protective response has not been fully elucidated. The present work aims at evaluating the participation of neutrophils in the immune response induced by P10+DODAB. We found that the vaccine reduced both the influx of pulmonary neutrophils and the fungal load in comparison to infected animals that did not receive this treatment. The parenchymal architecture of the lungs of P10+DODAB-treated animals was largely preserved with only a few granulomas present, and tissue cytokine analysis showed a Th1 cytokine profile with augmented levels of IL-12, IFN-γ and TNF-α, and low levels of IL-4. When neutrophils were depleted 24 h prior to each treatment, the effectiveness of the P10+DODAB vaccine was completely lost as the fungal burdens remained high and histological examination showed a marked inflammation and fungal dissemination with a dysregulated cytokine response. In conclusion, these findings indicate that neutrophils are vital to ensure the triggering of an effective immune response to P10+DODAB.

## 1. Introduction

Paracoccidioidomycosis (PCM) is a systemic mycosis that most frequently occurs in individuals in Brazil, Colombia, Venezuela, and Argentina [1]. Imported cases also occur in other areas in the Americas as well as in Europe and Asia. The disease is due to the thermally dimorphic fungi of the Paracoccidioides genus, specifically represented by two main species: *P. brasiliensis* and *P. lutzii* [2,3,4]. However, more recently, three other species were suggested: *P. restrepiensis*, *P. venezuelensis,* and *P. americana* [5]. Durations of treatment are long (frequently ~2 years) and relapses are common [6]. Additionally, resistance of *P. brasiliensis* to antifungals, like azole derivatives, may occur [7].

There is an urgent need for new therapeutic approaches that reduce the treatment time, relapses, and disease sequelae (especially fibrosis) in patients with PCM. One area of research has focused on the development of a vaccine against PCM. Of all the vaccine candidates, the most advanced is based on the peptide called “P10”, which is derived from the 43kDa glycoprotein of *P. brasiliensis* (gp43) [8]. In animal models, the administration of P10 to infected mice efficiently reduces the pulmonary fungal load, prevents the dissemination to other organs, and induces a Th1 immune response [8,9,10,11,12]. More interestingly, the therapeutic version of the P10-vaccine (administered after infection) has produced excellent results when associated with conventional chemotherapy regimens for PCM [13,14].

The investigation of the roles of different cell types in the protective dynamics of P10 treatment is essential for the further development of the vaccine. Neutrophils are important cells associated with protection and immune regulation during the initial phase of PCM [15]. However, these cells are detrimental in the chronic stage of the disease, as they are associated with fibrosis [16]. Hence, we sought to evaluate the role of neutrophils in a therapeutic vaccine against PCM.

## 2. Materials and Methods

### 2.1. Animals

Male BALB/c mice (6 to 8 weeks old; 6 animals/group) were housed in polypropylene cages under specific pathogen-free conditions. These animals were bred at the Animal Facility in the Institute of Biomedical Sciences of University of São Paulo. All experiments involving animals were conducted and approved by the Ethics Committee of the Institute (protocol number 044, page 127, book 2) and conducted in accordance with international recommendations.

### 2.2. Fungus Strain

Virulent *P. brasiliensis* Pb18 yeast cells were used to infect the animals. The strain was maintained by a weekly passage on agar Sabouraud dextrose (Sabouraud, BACTO^TM^, BD Franklin Lakes, NJ, USA) at 37 °C. For experiments in vivo or in vitro, 7–10 day-old yeasts were grown in brain heart infusion broth (BHI, BACTO^TM^, BD Franklin Lakes, NJ, USA) at 37 °C for 5–7 days under shaking (150 RPM). The yeast cells were washed in phosphate-buffered saline (PBS, pH 7.2) and counted in a hemocytometer. The viability of fungal suspensions was determined by staining with Trypan blue (Sigma-Aldrich, St. Louis, MO, USA) and was always higher than 90%.

### 2.3. Intratracheal (i.t) Infection

BALB/c mice were inoculated intratracheally with 3 × 10^5^ virulent Pb18 yeasts. Each mouse was inoculated a maximum volume of 50 µL. Briefly, mice were anesthetized intraperitoneally with a solution containing 80 mg/Kg of ketamine (União Química Farmacêutica, São Paulo, SP, Brazil) and 10 mg/Kg of xylazine (União Química Farmacêutica, São Paulo, SP, Brazil). After 5 min, their necks were hyperextended, the tracheas were exposed at the level of the thyroid, and yeast cells were inoculated with a tuberculin needle (27G). Incisions were sutured with 5–0 silk.

### 2.4. Peptide Synthesis and Purification

The P10 peptide (QTLIAIHTLAIRYAN) used in this study was purchased from Peptide 2.0 (Chantilly, VA, USA). HPLC and MS analyses showed that the synthetic P10 was 98% pure.

### 2.5. Dioctadecyl-Dimethylammonium Bromide Formulation

The cationic lipid *dioctadecyl-dimethylammonium bromide* [DODAB; Sigma-Aldrich, St. Louis, MO, USA) was prepared at the concentration of 2 mM in 1 mM NaCl. The solution was sonicated (80–90 mW, 20 min, 70 °C) in order to obtain bilayer fragments with median diameters of 73 ± 1 nm and zeta potentials of 41 ± 3 mV. The solution was then centrifuged (2000× *g*/30 min) and the supernatant was diluted in 1 mM NaCl to obtain the correct concentration for use as an adjuvant during immunizations [17].

### 2.6. Therapeutic Vaccine Schedule

Fifteen days after infection with *P. brasiliensis*, the mice were treated subcutaneously with a vaccine formulation consisting of peptide P10 and DODAB (P10-DODAB) in 1 mM NaCl (vehicle) at 20 µg of peptide and 0.1 mM of lipid per animal. This vaccine was administered subcutaneously at 7-day intervals between doses (i.e., vaccine was administered at days 15, 22, and 29). Seven days after the last dose (on day 36), the animals were euthanized, and the fungal load, cytokine profile, and lung pathology were evaluated between different groups. Controls included infected and untreated mice as well as animals that received only P10 or DODAB at the same intervals as those for P10-DODAB.

### 2.7. Neutrophil Depletion

**Figure 1:**
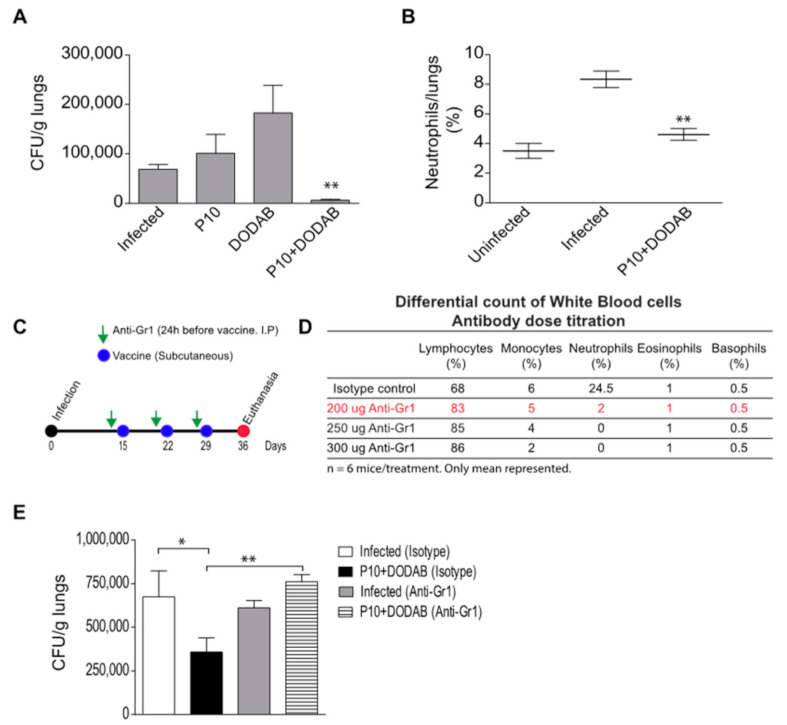
Effectivity of a therapeutic vaccine against paracoccidioidomycosis is associated with the presence of neutrophils in the immunization site. Mice were infected with *P. brasiliensis* (Pb18 strain), and after 15 days the animals were treated or not with a therapeutic P10+DODAB vaccine, P10 only or DODAB alone (three doses administered one week apart). The mice were euthanized 36 days after infection, and (**A**) the fungal load was determined by CFU and (**B**) the number of neutrophils in the lungs was measured by flow cytometry. In another model the neutrophils were depleted 24 h before each vaccination (**C**) and the fungal burden in the lungs was measured 36 days after infection. The optimal dose of antibody was previously titrated in uninfected and unvaccinated animals, and represented in (**D**). (**E**) The neutrophil depletion was achieved through the systemic (intraperitoneal route) administration of the anti-Gr1 antibody. Data are representative of three independent experiments (mean ± SD). * *p* ≤ 0.05, ** *p* ≤ 0.005, One-Way ANOVA test with the Tukey test for multiple comparison.

The mice were injected intraperitoneally with 200 µg of mAb anti-Gr-1 (clone RB6/8C5) 24 h before each dose of the therapeutic vaccine. The depletion effect lasted for 48 h after each administration (Figure 1D). Control mice were injected with an equivalent amount of an isotype control IgG2b (BD bioscience, San Diego, CA, USA). The anti-Gr-1 antibody was obtained from culture supernatants of hybridoma RB6/8C5. The antibody was purified by protein A/G column (Thermo Fischer Scientific, Waltham, MA, USA) and quantified by NanoDrop. The optimal dose of mAb anti-GR-1 was determined by the differential count of white blood cells using the Instant Prov kit (NewProv, Pinhais, PR, Brazil) after treatment of the mice with different amounts of antibodies (Figure 1C).

### 2.8. Lung Digestion and Flow Cytometry

The mice were euthanized using a CO2 chamber and the lungs were removed. The organ was washed with PBS, cut into small pieces (1 mm), and incubated with 0.5 mg/mL collagenase D (Roche Hungary Ltd., Budapest, Hungary) at 37 °C under shaker (200 rpm) for 45 min. The reaction was stopped with FBS and the suspension was dispersed with 10 mL syringe and needle (18G). The suspension was filtered in a 70 µm cell strainer and spun down (300× *g*/10 min at 4 °C). Red blood cells were lysed with 0.2% NaCl and then stopped with an equal volume of 1.6% NaCl. The cells were washed with complete RPMI (10% FBS and 10 µg/mL gentamicin) and counted in a hemacytometer. Trypan Blue was used to evaluate the viability. A total of 3 × 10^6^ cells were washed with FACS buffer (0.5% BSA, 2 mM EDTA) and incubated with Fc Block (BD, USA) for 10 min at room temperature. The cells were washed and stained with the following antibody (0.5 µg/10^6^ cells) cocktail for 30 min at 4 °C: PE anti-CD45 (clone 30-F11. BD, USA), AlexaFluor 700 anti-CD11b (clone M1/70. eBioscience, San Diego, CA, USA), and PerCP-Cy5.5 anti-Ly6G (clone 1A8. BD, USA). The cells were washed and fixed with 1% paraformaldehyde (in PBS, pH 7.2). A total of 100.000 events were acquired per sample. The LSR Fortessa cytometer (BD, USA) and FlowJo software (v. 10, ThreeStar Inc., Ashland, VA, USA) were used to acquire the events and to perform the analysis, respectively.

### 2.9. Histopathology and Pulmonary Fungal Load

For each mouse, a randomized part of the lungs was excised, fixed in 10% buffered formalin (Merck, Darmstadt, Germany), stained with hematoxylin and eosin (H&E), and evaluated by light microscopy. The rest of the lungs was weighed, individually homogenized in 2 mL of sterile PBS, and 100 µL of this suspension was plated on agar BHI supplemented with 4% fetal bovine serum (Gibco, Gaithersburg, MD, USA), 5% spent culture medium of *P. brasiliensis* 192 isolate, and streptomycin/penicillin 100 IU/mL (Cultilab, Campinas, Brazil). The plates were incubated at 37 °C for a period of 10 days. The numbers of colonies were counted, and the results were expressed in colony forming units (CFU) per gram of tissue.

### 2.10. Cytokine Analysis

500 µL of lung homogenate (see Section 2.8) was mixed with an equal part of protease inhibitors cocktail (Sigma-Aldrich, St. Louis, MO, USA), centrifuged, and the clear supernatant was kept at −20 °C until further analysis. Commercial ELISA kits (BD Biosciences, San Diego, CA, USA) were used to quantify supernatant levels of IL-4, IL-10, IL-12, TNF-α, IFN-γ, MCP-1, and GM-CSF. Similarly, IL-1β was quantified with a kit from eBioscience, USA. The results were expressed in pg/mL.

### 2.11. Statistical Analysis

Statistics were performed using Prism 5. The Shapiro–Wilk test was used to analyze the normal distribution of the data. The results were expressed as means and standard deviations (SDs) of the indicated values. The One-way ANOVA test was employed to identify significant differences between groups. The Tukey test was used for post-hoc analysis. *p*-values of ≤ 0.05 indicated statistical significance.

## 3. Results

### 3.1. Pulmonary Fungal Load

The association of a known Th1-epitope harboring peptide (P10) from a *P. brasiliensis* gp43 molecule with a cationic lipid (DODAB) significantly reduced the pulmonary fungal load in mice infected with *P. brasiliensis* (Figure 1A). The treatment with DODAB alone increased the fungal loads in the mice, but the difference was not statistically significant. We also investigated the correlation between vaccine treatment, fungal load, and the presence of neutrophils in situ (lungs). Notably, the infected mice treated with P10+DODAB had a significant reduction in neutrophil numbers in the lungs compared to untreated, infected mice (Figure 1B). In addition, the pulmonary neutrophil levels in the P10+DODAB treated, infected mice were similar to those found in uninfected animals. In order to establish the role of neutrophils in this infection dynamic and vaccine efficacy, we depleted neutrophils before each vaccination and evaluated the impact on the fungal load (Figure 1C). Interestingly, the depletion of neutrophils abolished the protective effects of P10+DODAB (Figure 1E).

### 3.2. Histopathology

**Figure 2:**
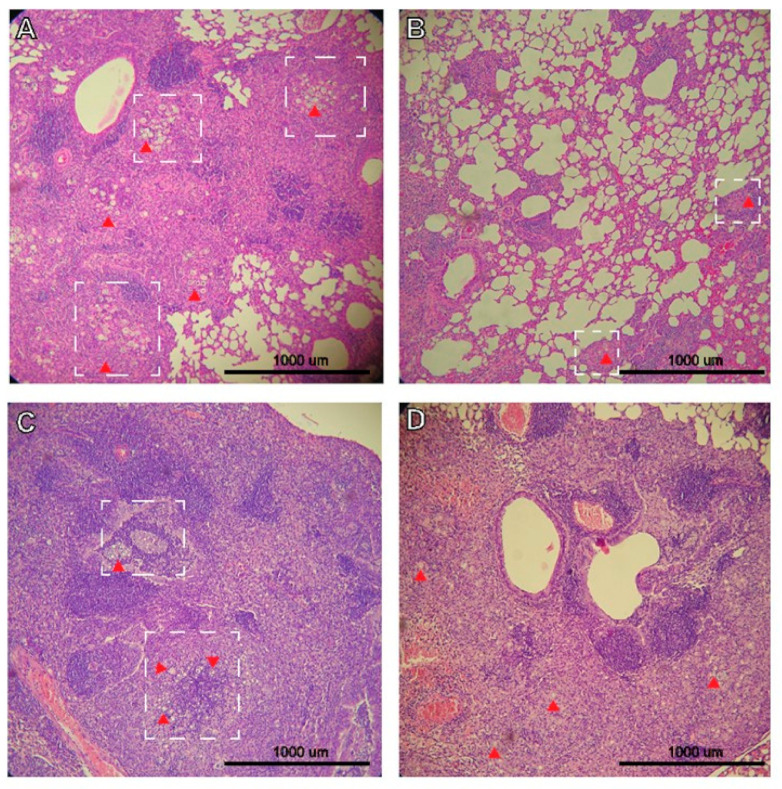
Impact of neutrophils on the lung inflammatory response. Infected mice were given an IgG2b isotype control on days 14, 21, and 28 post infection, and either PBS (**A**) or P10+DODAB (**B**) on days 15, 22, and 29 post infection with P10+DODAB. Another set of mice were infected and given neutrophil-depleting antibody Gr1 on days 14, 21, and 28 post infection, and either PBS (**C**) or P10+DODAB (**D**) on days 15, 22, and 29. Mice were euthanized 36 days post infection and the inflammatory response in the lungs was evaluated by histology using hematoxylin/eosin stain. 100× magnification. Red arrow = *P. brasiliensis* yeasts; dashed white square = area with high concentration of yeast/granuloma formation.

The lungs of mice infected with *P. brasiliensis* displayed extensive inflammation and diffuse yeasts (Figure 2A). However, treatment with P10+DODAB resulted in the overall maintenance of the pulmonary architecture with markedly decreased inflammation, and yeast cells were restricted to granulomas (Figure 2B). However, P10+DODAB treatment of the mice that received the neutrophil-depleting antibody failed to decrease the pulmonary inflammatory process or control the growth of *P. brasiliensis* yeasts (Figure 2D).

### 3.3. Cytokine Analysis

**Figure 3:**
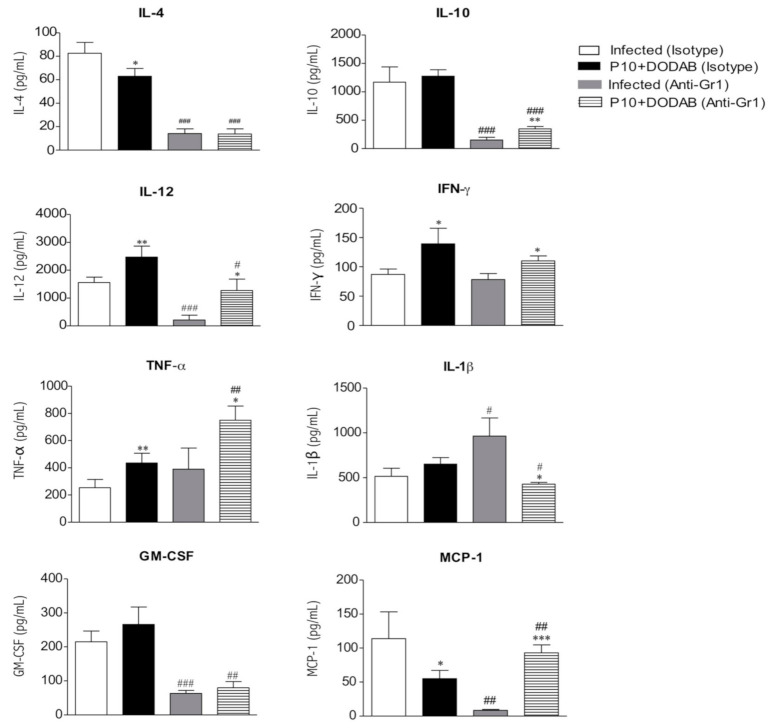
Neutrophil depletion before therapeutic vaccine impacts cytokine production. Infected mice were untreated and given neutrophil-depleting antibody Gr1 [Infected (anti-Gr1)] or an IgG2b isotype control [Infected (Isotype)] at days 14, 21, and 28 post infection. Additional mice were infected and given neutrophil-depleting antibody Gr1 or an IgG2b isotype control at days 14, 21, and 28 post infection, and were vaccinated at days 15, 22, and 29 post infection with P10+DODAB (P10+DODAB (anti-Gr1) and P10+DODAB (Isotype), respectively). Mice were euthanized 36 days post infection and the cytokine levels in the lungs were quantified. Data are representative of three independent experiments (mean ± SD). Statistical comparison by One-way ANOVA related to depletion (Infected (Isotype) vs. P10+DODAB (Isotype) or infected (anti-Gr1) vs. P10+DODAB (anti-Gr1)): * *p* ≤ 0.01, ** *p* ≤ 0.005, and *** *p* ≤ 0.0001; or vaccine (Infected (Isotype) vs. Infected (Anti-Gr1) or P10+DODAB (Isotype) vs. P10+DODAB (Anti-Gr1)): ^#^ *p* ≤ 0.01, ^##^ *p*≤ 0.005, ^###^ *p*≤ 0.0001.

The P10+DODAB vaccination resulted in an increase in IL12, IFN-γ, and TNF-α as well as a reduction in IL-4 and MCP-1 compared to the levels found in infected, untreated mice (Figure 3). Neutrophil depletion resulted in the reduction of IL-4, IL-10, IL-12, GM-CSF, and MCP-1 as well as an increase in IL-1β relative to those in infected, untreated mice. However, the P10+DODAB-vaccinated, neutrophil-depleted mice had increased amounts of IL-10, IL-12, GM-CSF, MCP-1, IFN-γ, and TNF-α as well as lower levels of IL-β compared to infected, neutrophil-depleted mice. Moreover, these P10+DODAB-vaccinated, neutrophil-depleted mice had lower levels of IL-4, IL-10, IL-12, IL-1β, and GM-CSF as well as higher amounts of MCP-1 and TNF-α relative to P10+DODAB-vaccinated mice that did not receive the neutrophil-depleting antibody.

## 4. Discussion

Neutrophils have been recognized as important players in adaptive immune responses, especially in the context of vaccine responses [18,19,20,21,22]. Our findings demonstrate that neutrophils are essential for the protective effects of a therapeutic vaccine against PCM. Mice that received the therapeutic vaccine had a significantly reduced fungal burden and neutrophil levels similar to those of uninfected controls, whereas neutrophil levels were significantly elevated in unvaccinated infected mice. A previous study demonstrated that immunosuppressed mice immunized with P10 and infected with *P. brasiliensis* developed a robust infiltration of Ly-6C/Ly-6G+ cells (presumably neutrophils) around compact granuloma with few viable yeasts inside [23]. The experimental design in this prior study and the currently used approach are different. Both studies, however, confirm the dual role of neutrophils during the PCM [15,16], and also show that P10-based vaccine can promote the increase or decrease of these cells in the target tissue, depending on the biology of the host. Another important inference is that quality can be more important than quantity as neutrophils do not necessarily need to be amplified in the tissue, but rather function in a specific activation state [24,25]. Further studies are needed to further characterize these variations in different host conditions.

The present work demonstrated that the efficacy of the P10+DODAB vaccine was completely lost in the absence of neutrophils at the time of vaccinations. This suggests that neutrophils need to be recruited to the area where the subcutaneous vaccine is applied in order to capture antigen and present it to T cells in secondary lymphoid organs. This hypothesis is consistent with results by other groups with different organisms and models [20,26,27,28]. In addition, we saw that the production of cytokines was completely altered in the depleted mice, which shows that neutrophils have a modulatory role in the overall immune response [18,20]. This is consistent with a previous publication that found that neutrophil depletion can change cytokine dynamics in PCM infection [29]. In contrast, the altered cytokine production could be a result of reduced ingestion of apoptotic neutrophil by macrophages, an event that has been described as important in cytokine production [30,31,32].

The resolution of PCM relies on the Th1 immune response, where the yeasts are efficiently recognized, engulfed, and killed by macrophages [33,34]. In this context, the P10-based vaccine confers protection or reduces the fungal load by inducing the production of Th1-signature cytokines, such as IL-12 and IFN-γ [8,13,23,35,36,37]. Animals treated with the P10+DODAB vaccine showed a pulmonary parenchyma that was markedly less inflamed, containing relatively low numbers of compact granulomas with few yeasts. In contrast, neutrophil-deficient animals displayed severe pulmonary inflammatory alterations with yeasts disseminated throughout the tissue, with or without P10+DODAB administration. The Th1 immune response is still present in the neutrophil-depleted animals that received P10+DODAB, but the increase in TNF-α and MCP-1 levels led to the potent inflammation in situ. TNF-α increases the macrophage killing capacity by inducing the production of nitric oxide (NO), a potent microbicide [38,39,40]. MCP-1 is a key chemokine that regulates the migration of macrophages to the tissue [41,42]. Thus, the depletion of neutrophils before each vaccination induced a brisk migration of macrophages to the tissues resulting in an exuberant response that appears to be harmful to the host. In addition, this uncontrolled inflammation was also favored by low levels of IL-10 in these animals, an important regulatory cytokine [43].

A critical issue that may impact our overall conclusion is the use of antibody to Gr1 instead of the antibody targeting Ly6G to deplete the neutrophils in the mice. The antibody to Gr1 can potentially deplete other cell subsets such as dendritic cells and monocytes, while the antibody to Ly6G is more specific for neutrophils [44,45]. With the dose used, however, neutrophils were depleted but other cells were preserved, as demonstrated by a differential count of cells in the blood. Still, we cannot exclude the possibility that other cells have a contribution to our model. Future studies should continue to explore the roles of different host effector cells in our PCM/vaccine model. In addition, for better understanding, it is important to demonstrate the impact of neutrophils on different routes of vaccination.

## Data Availability

Not applicable.
